# Efficacy of electroacupuncture for the treatment of asthenozoospermia: A protocol for systematic review and meta-analysis

**DOI:** 10.1097/MD.0000000000023350

**Published:** 2021-01-29

**Authors:** Xinhui Wu, Di’ang Chen, Yexin Zhou, Ting Xia

**Affiliations:** a Hospital of Chengdu University of Traditional Chinese Medicine, Chengdu, Sichuan Province; b Guangxi University of Chinese Medicine, Nanning, Guangxi Province, China.

**Keywords:** asthenozoospermia, electroacupuncture, protocol, systematic review

## Abstract

**Introduction:**

Infertility has affected millions of couples aged 15 to 44 years worldwide. Recently, some studies suggest that abnormal semen quality is the main cause of male infertility and asthenozoospermia accounts for 19% of the infertility of men. The situation has brought a huge burden to the patient with asthenozoospermia and society. Acupuncture is a part of traditional Chinese medicine. Electroacupuncture (EA) has gained in popularity. Although a positive effect of manual acupuncture and EA on sperm parameters has been documented in several studies, there still a lack of more solid evidence. We hope to provide a convincing study for EA.

**Methods and analysis:**

The electronic databases of MEDLINE, PubMed, Web of Science, EMBASE, Cochrane Library, Clinicaltrials. org, China National Knowledge Infrastructure Database (CNKI), Wan fang Database, China Biology Medicine Database (CBM), VIP Science Technology Periodical Database, Chinese Clinical Trial Registry will be retrieved. All the randomized controlled trials of rESWT for patients with CP/CPPS will be included. We will evaluate the outcomes including NIH-CPSI, VAS, IPSS, IIEF-5, and conduct this study strictly according to the Cochrane Handbook for Systematic Reviews of Interventions.

**Results:**

The present study is a protocol for systematic review and meta-analysis without results, and data analysis will be carried out after the protocol. We will share our findings on October 31st, 2021.

**Conclusions:**

EA for asthenospermia is a microtrauma surgery with less pain. EA can effectively improve sperm motility; however, its efficacy has not been assessed scientifically and systematically. To address this limitation, this study will inspect the efficacy and safety of the EA in patients with asthenospermia.

**Ethics and dissemination:**

Formal ethical approval is not required in this protocol. We will collect and analyze data based on published studies, and since there are no patients involved in this study, individual privacy will not be under concerns. The results of this review will be disseminated to peer-reviewed journals or submit to related conferences.

**Protocol registration number:**

INPLASY2020100071

## 1 Introduction

Infertility is a disease of the reproductive system defined by the failure to achieve a clinical pregnancy after ≥12 months of regular unprotected sexual intercourse which has become a worldwide problem.^[1]^ According to the epidemiology, infertility has affected millions of couples aged 15 to 44 years,^[2]^ and the factor of male account for nearly half.^[3]^ Recently, some studies suggest that abnormal semen quality is the main cause of male infertility^[4]^ and asthenozoospermia accounts for 19% of the infertility of men.^[5]^ The situation has brought a huge burden to the patient with asthenozoospermia and society.

As the etiology and pathogenesis of asthenospermia are complicated, there still are some arguments. In the past several decades, most of the studies of asthenospermia's treatment are focused on the regulation of hormones,^[6]^ antioxidation,^[7,8]^ providing energy for sperm,^[9,10]^ suppling trace elements,^[11,12]^ and assisted reproductive technology (ATR).^[13]^ However, the efficacy of a single drug is not satisfying, it needs to be combined in clinical treatment. Moreover, hormone drugs have some unclear side effects, and ATR costs a lot of money, the situation puts forward higher requirements for the treatment of asthenospermia. A few studies have shown that varicocelectomy can be performed for the patient with asthenospermia and varicocele.^[14,15]^ Chinese scholars suggested^[16]^ that acupuncture improves the sperm motility of patients with asthenospermia.

Acupuncture is a part of traditional Chinese medicine that has been used to treat various disorders in the East for more than 3000 years and is now gaining widespread acceptance. Comparing with traditional manual acupuncture (MA), in recent decades new acupuncture modalities such as electroacupuncture^[17]^ (EA) have gained popularity. Although a positive effect of MA and EA on sperm parameters has been documented in several studies, there is still a lack of more solid evidence. We hope to provide a convincing study for EA.

## 2 Objectives

The purpose of this study is to further evaluate the effectiveness and safety of EA in the treatment of asthenospermia. The results will provide urologists and andrologists with clinical surgery decisions.

## 3 Methods

The protocol was registered on the International Platform of Registered Systematic Review and Meta-analysis Protocols (registration number: INPLASY2020100071) which could be available on *https://inplasy.com*. The content refers to the statement of the Preferred Reporting Items for Systematic Review and Meta-Analysis Protocols (PRISMA-P) checklist.^[18]^

### 3.1 Eligibility criteria

The inclusion and exclusion criteria are as follows.

#### 3.1.1 Types of studies

All the RCTs of EA for patients with asthenospermia will be included without publication status restriction or writing language letters to editors, review articles, case reports, conference abstracts, cross-sectional studies, and all observational studies will be excluded.

#### 3.1.2 Participants

##### 3.1.2.1 Inclusion criteria

- Diagnosed as asthenospermia based on WHO reference values.
- The diagnosis was confirmed by at least 2 consecutive semen analyses performed on ejaculates collected after 3–7 days of sexual abstinence.

##### 3.1.2.2 Exclusion criteria

- Patient with ejaculatory dysfunction.
- Patients with a medical history of risk factors for infertility (eg, prior vasectomy, orchidopexy, or varicocele).
- Patients with immune-related infertility.
- Patients with occupational exposure to agents suspected to impact male reproduction.
- Patients with a history of previous infertility treatment (eg, hormonal therapy).
- Patients with genetic disease.

#### 3.1.3 Types of interventions and controls

##### 3.1.3.1 Experimental interventions:

The patients in the treatment group received EA.

##### 3.1.3.2 Control interventions:

The control group could gain hormonal therapy, antioxidant therapy, trace elements, energy support therapy, or guideline-recommended conventional treatment.

#### 3.1.4 Types of outcome measures

##### 3.1.4.1 Primary outcome:

1. Sperm motility.

##### 3.1.4.2 Secondary outcomes:

1. Sperm DNA fragmentation index (DFI)

### 3.2 Search strategy

#### 3.2.1 Data sources

The electronic databases of MEDLINE, PubMed, Web of Science, EMBASE, Cochrane Library, Clinicaltrials. org, China National Knowledge Infrastructure Database (CNKI), Wan fang Database, China Biology Medicine Database (CBM), VIP Science Technology Periodical Database, Chinese Clinical Trial Registry will be retrieved. They will be searched until October 2021 to recognize related studies. The search strategy that will be run in the PubMed and adjusted to fit the other database when necessary is presented in Table 1.

**Table 1:** Example of PubMed search strategy.

Number	Search terms
#1	Acupuncture Points [Mesh]
#2	Acupuncture Point [All Fields] OR Point, Acupuncture [All Fields] OR Points, Acupuncture [All Fields] OR Acupoints [All Fields] OR Acupoint [All Fields]
#3	Electroacupuncture [Mesh]
#4	Asthenozoospermia [Mesh]
#5	Astheno Teratozoospermia [All Fields] OR Astheno Teratozoospermias [All Fields] OR Teratozoospermia, Astheno [All Fields] OR Teratozoospermias, Astheno [All Fields] OR Asthenoteratozoospermia [All Fields] OR Asthenoteratozoospermias [All Fields]
#6	#1 OR #2 OR #3
#7	#4 OR #5
#8	#6 AND #7

#### 3.2.2 Other sources of search

Gray literature will be retrieved through Open Grey. Besides, we will also scan the reference lists of manual review articles for any possible titles matching the inclusion criteria.

### 3.3 Data extraction, quality, and validation

#### 3.3.1 Study inclusion

**Figure 1:**
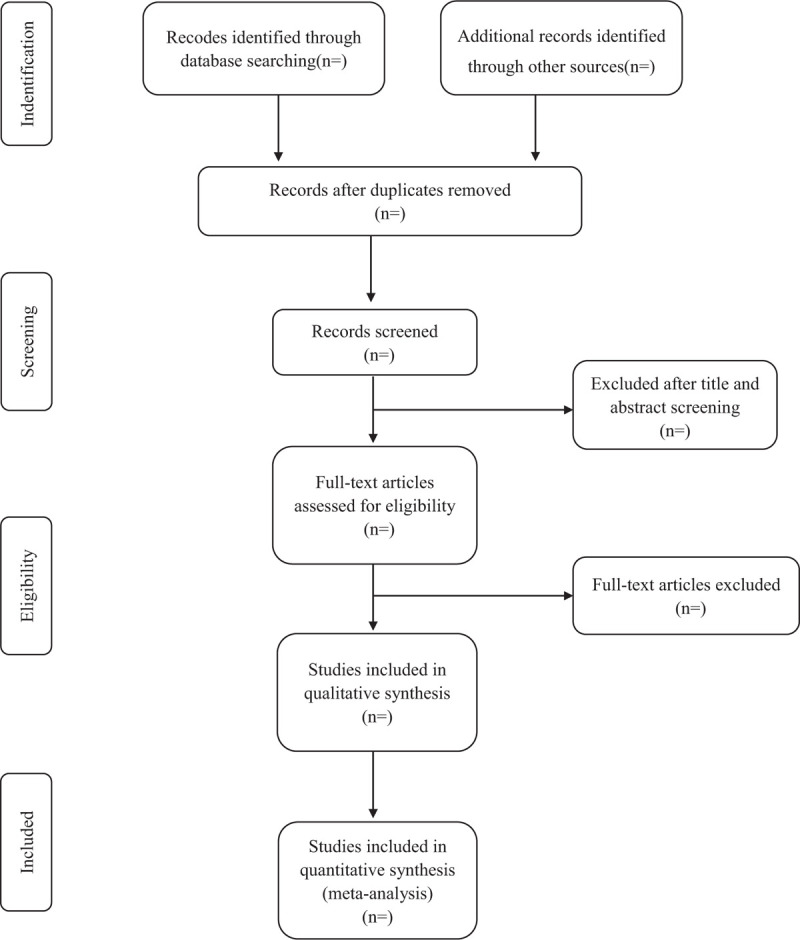
Study selection flow chart.

According to predefined eligibility criteria, importing the literature retrieved to the Endnote X8 and eliminate the duplicate data. The software will be used to filter duplicate documents first, and then the studies which do not meet the inclusion criteria will be removed. If the studies appear to meet the inclusion criteria or there is any uncertainty based on the information provided in the title and abstract, full texts will be obtained for further assessment. Further detailed screening and data extraction of the documents will be performed simultaneously by 2 professionally trained reviewers. When necessary, the original study author will be contacted for judgment. Disagreements will be resolved by discussion or taking the expert (DAC) for arbitration. The number and reasons for excluding trials will be recorded in detail. A flow diagram of the study selection is shown in Figure 1.

#### 3.3.2 Data extraction and management

The 2 reviewers will independently read and extract the data from the study. Before the formal process of data extraction, the review group will discuss and a unified data extraction form (an excel spreadsheet) will be produced. The content data will include the following information: title, abstract, first author and corresponding author, the country, the publishing year, publications, participants, demographic characteristics (age, family situation, regional, ethnic, and national), the number of participants, diagnostic criteria, types, intervention, observation index (sperm motility, DFI), the results of the study, the incidence of adverse events and type. All disagreement between the 2 reviewers will be decided by consensus or with the participation of a third reviewer. Besides, we will also contact the author via email to request any missing data or clarification. If we cannot obtain the missing data, we will report it in the risk assessment of bias and consider its impact on the analysis of the data.

### 3.4 Risk of bias assessment

We will evaluate selection bias, detection bias, attrition bias, performance bias, and other biases based on the Cochrane Collaboration Network Risk Assessment Tool. The tool assesses the risk of bias mainly in the following 7 aspects: random sequence generation, allocation concealment, the blinding method for patients, researchers and outcomes assessors, incomplete result data, and selective reports. As recommended by the Cochrane manual, the risk of bias in each of these areas will be assessed as low or high depending on whether the criteria were met or not met, and the lack of information will be recorded as unclear. The risk of bias will be checked by 2 review authors. Discrepancies between review authors on the risk of bias will be resolved through discussion with a third review author.

### 3.5 Quantitative data synthesis and statistical methods

#### 3.5.1 Data analysis and synthesis

The RevMan5.3 software will be used to conduct the meta-analysis (If feasible). Descriptive analysis or systematic narrative synthesis will be performed to summarize and explain the characteristics and findings of the included studies and provide the information in the texts and tables. For dichotomous data (eg, effective and ineffective), we will calculate risk ratio (RR) and 95% confidence intervals (CIs). For continuous data, which will be pooled as mean difference (MD).

#### 3.5.2 Investigation of heterogeneity

The Q statistic and *I*^*2*^ statistic of Cochran will be used for testing heterogeneity. If *P* ≤ .10 or *I*^*2*^ ≥ 50%, heterogeneity will be considered significant. At this point, a fixed-effects model (Mantel-Haenzel method for RR and Inverse Variance for MD) will be used for *I*^*2*^ < 50%. A random-effects model (D-I method) will be used when the heterogeneity is still significant after sensitivity analysis and subgroup analysis.

#### 3.5.3 Subgroup analysis

If necessary, we will identify the source of heterogeneity through subgroup analysis and manage the heterogeneity:

1. The duration and severity of asthenospermia.
2. Demographic characteristics of the patients: age, marital and family status, region, race.
3. Therapy time.
4. Follow-up time.

#### 3.5.4 Sensitivity analysis

Sensitivity analysis will be used to test the reliability and stability of the meta-analysis results, and to assess the source of heterogeneity. We will compare the results before and after by excluding trials with a high risk of bias or eliminating each study individually 1 study each time and then pooling the remaining studies.

#### 3.5.5 Grading the quality of evidence

The GRADE tool^[19]^ will be applied to judge the quality of evidence in the systematic review. It consists of risk of bias, consistency, directness, precision, and publication bias. Two independent reviewers will assess these studies. In most cases, disagreements were resolved by discussion between the 2 reviewers. If disagreement remained after discussion, the third reviewer will be consulted before taking the final decision on the disagreements.

#### 3.5.6 Publication bias

Published bias will be measured by the funnel plot. If the result is indistinct, the Begg test and Egger test will be used (by STATA software 11.0).

#### 3.5.7 Reporting of the review

The quality of the manuscript will be standardized by each item of the AMSTAR-2 tool. And the results will be reported following the Preferred Reporting Items for Systematic Reviews and Meta-Analysis (PRISMA) statement.^[20]^

## 4 Discussion

EA for asthenospermia is a microtrauma surgery with less pain. So, it is crucial to make sure whether EA is a good option for patients. The previous studies have indicated that EA can effectively improve sperm motility; however, its efficacy has not been assessed scientifically and systematically. To address this limitation, this study will inspect the efficacy and safety of the EA in patients with asthenospermia. This review also has some limitations. The different acupoints of EA and the different severity of asthenospermia may induce the heterogeneity.

## Author contributions

**Conceptualization:** Xinhui Wu, Diang Chen

**Data curation:** Xinhui Wu, Diang Chen, Yexin Zhou, Ting Xia

**Formal analysis**: Xinhui Wu, Diang Chen

**Methodology:** Xinhui Wu, Diang Chen, Ting Xia.

**Project administration:** Xinhui Wu, Yexin Zhou

**Software:** Diang Chen, Ting Xia

**Supervision:** Xinhui Wu, Diang Chen, Yexin Zhou

**Validation:** Diang Chen, Yexin Zhou

**Writing – original draft:** Xinhui Wu, Diang Chen

**Writing – review & editing:** Xinhui Wu, Diang Chen, Yexin Zhou

DAC is the guarantor. All authors read, provided feedback, and approved the final manuscript.

## Abbreviations

Abbreviations: ATR = assisted reproductive technology, CBM = China Biology Medicine Database, CNKI = China National Knowledge Infrastructure Database, DFI = Sperm DNA fragmentation index, EA = electroacupuncture, MA = manual acupuncture, MD = mean difference, RR = risk ratio.
